# Accounting for uncertainty in forest management models

**DOI:** 10.1016/j.foreco.2020.118186

**Published:** 2020-07-15

**Authors:** Francesca Rinaldi, Ragnar Jonsson

**Affiliations:** European Commission Joint Research Centre (JRC), Via E. Fermi, 2749 I-21027 Ispra, Italy

**Keywords:** Forest management, Harvesting decision, Uncertainty, Climate change, Information, Control theory

## Abstract

Forests and their ecosystem services are subjected to uncertain factors, causing drastic changes in forest production and/or market conditions, the impacts of which cannot be precisely estimated beforehand. We introduce a theoretical framework, based on control theory, for robust optimization of forest management under uncertainty. Forest owners herein regard their decision support system only as an approximation to an unknown, true, model. Concerns about model misspecification incite them to seek the single harvesting rule that works well over a set of models statistically similar to their approximation. Accounting for mistrust of decision support systems in modelling harvesting behavior is particularly relevant in view of uncertainty induced by climate change. We use a stylized forest model to explore the effects of uncertainty on harvesting decisions, also considering the role of information aimed at reducing, or making forest owners aware of, such uncertainty. The simulation results demonstrate that model uncertainty affects harvesting intensity and thus forest development. Harvest levels are lower for forest managers concerned with model uncertainty, favoring stand volume over harvest revenues. Further, information release affects the level of perceived uncertainty and thus harvesting behavior and forest development, reaffirming the importance of information as a forest policy instrument.

## Highlights

• Concern for the accuracy of decision support systems affects harvesting behavior.
• We introduce a model accounting for uncertainty as to DSS accuracy.
• Model uncertainty concerned managers favor stand volume over harvest revenue.
• Information affects the level of perceived uncertainty and thus harvest behavior.

## 1 Introduction

Uncertain factors are continuously affecting the functionality of ecosystems and, thus, the provision of ecosystem services. Notably, the still rather weak understanding of the magnitudes of climate change—if not the general direction—and even more the manifestations thereof and how forest ecosystems will react, creates considerable uncertainty and poses challenges for forest managers (Park et al., 2014) and natural resource management in general (see, e.g., Aplet and McKinley, 2017). The forest sector—providing traditional products and bioenergy as well as new forest-based products—plays an increasingly crucial role in the bioeconomy (Ollikainen, 2014). Consequently, objectives and attitudes of forest owners/managers—expected to supply increasing volumes of woody biomass (see, e.g., Rinaldi and Jonsson, 2013)—are crucial, particularly those that govern harvest behavior.

Risk has been extensively debated in forest economics. Research has focused on deriving the optimal rotation age when landowners are risk-neutral—exceptions to risk-neutrality are Clarke and Reed, 1989, Alvarez and Koskela, 2007, Lien et al., 2007—and risk derives from uncertain future market perspective (Brazee and Mendelsohn, 1988, Willassen, 1998, Insley, 2002, Chang, 2005), or the possible arrival of a natural event compromising harvests (Englin et al., 2000, Amacher et al., 2005, Busby et al., 2013). All these studies implicitly assume that risk can be precisely modeled, meaning that the probability distribution characterizing any random event (e.g., the occurrence of a natural disaster, or the future wood price) is known in advance.

This assumption does not reflect reality, as the unique and full modeling either of the impact and the magnitude of a possible extremal shock affecting forest stand development (and the provision of related amenities) or the market conditions for forest products is concretely not possible. Thus, the forest growth and yield model used to describe the possible evolution of the stand after the shock becomes uncertain. In particular, Amacher (2015, p. 40) identifies uncertainty as “…cases where the forest landowners do not even know the distribution describing states of nature for market parameters, natural events, or their forest stocks”. Hence, the word ‘risk’ should be used to describe cases in which the realization of future variables cannot be determined in advance, but the distribution governing these realizations is known. This is the case, for example, when the future wood price is not known in advance, but it is assumed to follow a specific distribution with unique and certain mean and variance. In these cases, it is possible to define an expected value of the utility of the forest owner, as he knows the probabilities of any occurrence affecting the forest, and he acts based on that information. ‘Uncertainty’, instead, refers to cases for which the random distribution over the possible states of nature is not known in advance. It is important to notice that uncertainty does not exclusively refer to possible negative shocks—e.g., a negative demand shock and ensuing falling roundwood prices resulting from a large outflow of timber from salvage logging (as that caused by bark beetle infestation in Central Europe in 2019)—but also positive ones, e.g., unforeseen positive forest growth effects related to climate change (CO_2_ fertilization, longer vegetation periods) as well as positive demand shocks.

In forestry, uncertainty can affect forest landowner decision in many instances. First, the future distribution of market parameters might be unknown when landowners are to decide upon land-use or forest management decisions. Second, the forest stand, once established, is subject to the arrival of catastrophic natural events, whose magnitude and frequency cannot be a priori described by a single probability distribution. Finally, forest stand growth and associated amenities might follow unknown paths.

In this paper, we will focus on the second and third instances. Harvest behavior models typically assume that forest owners/managers fully trust the forest growth and yield model used for decision support in accurately representing the dynamics of their forest stands. Therefore, standard economic procedures, based on the assumption that the model is true (see, e.g., Koskela, 1989, Ollikainen, 1993, Uusivuori, 2002, Gong and Löfgren, 2003, Rinaldi and Jonsson, 2013), are used. However, predictions of this type of model frameworks are necessarily imprecise due to model misspecifications. This is a well-known problem for any modeler, often mistrusting his/her own model while calibrating it or performing specification tests. Therefore, the most straightforward reason for including the fear of model misspecifications in a theoretical framework is that, if modelers face specification doubts, so might the modeled decision makers (such as forest owners/managers in a harvest behavior model). This is not the least relevant in view of climate change, with forest growth models traditionally having been based on assumptions of climates and disturbance regimes varying within narrow, historically understood boundaries (Park et al., 2014), or even implicitly that local climate conditions will remain constant (Guariguata et al., 2008).

Thus, we introduce and demonstrate a theoretical framework wherein forest owners regard the forest growth and yield model used for decision support only as an approximation to an unknown model governing forest growth. Specifically, they assume that any model which is sufficiently (statistically) similar to their approximation might be the true growth model. We maintain that this particular representation could be sui for representing, in a stylized way, the effect of any natural shock on forest growth. Firstly, because the magnitude of the shock itself is unknown, second because different combinations of increases in temperature, precipitation, and CO_2_ concentration do not have a unique effect on plant growth. Additional uncertainty could possibly be induced by a general lack of information regarding extreme events, for which empirical knowledge and extended databases are not available due to the rarity of such events.

The next section introduces the theoretical framework. It describes the model used to represent forest growth, as well as the optimization problem for two representative forest owners, one who is unconcerned and another one who is concerned with model uncertainty. In the subsequent section, we conduct numerical analysis to test our set-up. The final section concludes the paper, discussing policy implications of the analysis and outlining possible future research developments.

## 2 The framework

We follow the methodology used in Hansen and Sargent (2007), wherein robust control techniques are adapted to acknowledge misspecification in economic modeling. Control theory has been widely applied to economic problems characterized by multi-stage decision processes. More specifically, optimal control of any economic problem, aimed at attaining a desired objective, can be defined as a problem of dynamic optimization, wherein an intertemporal objective function is maximized with respect to specific *control variables*, in order to satisfy a number of intertemporal constraints defined over a set of *state variables*. The solution to this problem involves finding an optimum for the admissible control variables, and then apply them to the system’s dynamics to derive the optimal trajectory of state variables. Classical control theory assumes that the dynamics governing the intertemporal transitions of state variables (the so-called model) are well known in advance by the decision maker (and by the modeler), and, furthermore, they are correctly specified.

However, model misspecification is a problem in economics, and this is why robust control came to the fore. Robust control theory considers a (dynamic) model only as an approximation to an unknown and unspecified model actually governing the process (or generating the data in econometrics applications). Therefore, a decision maker should seek *robust* decision rules and estimators that work over a continuous set of models near an approximating one, which is no longer considered true, but rather plausible, and which is taken only as a reference model. A closely related concept in economic theory is (knightian) uncertainty, relating to situations in which the decision maker describes the possible states of nature not by a unique probability distribution, but rather through a range of plausible probability distributions. In these situations, decisions cannot be made using the standard maximizing utility rule.^1^ Hansen and Sargent (2007) builds the bridge between (robust) control theory and (knightian) uncertainty suggesting the use of robust control methods to account for uncertainty, and to find the decision that works well over a set of possible deviations from the model used as reference.

In our framework—acknowledging the possibility for model misspecification—forest owners seek the single harvesting rule that works well, not only for the approximating model, but also over a set of models statistically similar to their approximation, one unknown element of which is considered to be the true growth model. Alternatively, one might think that a representative forest owner wants to put a lower bound on the performance of his harvesting decision, meaning that he tries to make good decisions when his model approximates the correct one. The harvesting decision is clearly related to the magnitude of the set of models the forest owner is considering as alternatives to the approximating one. This in turn depends on the degree of adverseness to uncertainty of the forest owner, or, alternatively on his/her degree of confidence in his approximating model. Specifically, the more averse, the less confident, he/she is, the higher the degree of precaution and the larger the set of alternative models considered. Forest owners represent a heterogeneous category in terms of objectives (see, e.g., Rinaldi et al., 2015), but also as to the degree of knowledge regarding the biological properties of their stands. The suggested framework allows the inclusion of such heterogeneity, by considering an approximating model (or decision support system) common to all agents,^2^ but characterizing them with different agent-specific degrees of mistrust of that model.

In the following, we will consider a single representative forest owner with an unspecified quadratic objective function, whose forest stand is assumed to grow according to standard linear dynamics. We expressly assume that the utility derives from the revenues he/she can obtain from his/her stand—and thus implicitly from the number of cut trees—as well as from the condition of the stand itself after harvesting—and thus from the number of standing trees. Specifically, let *u(y_t_,h_t_)* be the time dependent forest owner goal function evaluated at any time*)*. *y_t_* describes the general stand state at a given point in time *t*, with *y_it_* being the number of trees per acre in size-class *i* at time *t*. *h_t_* is a vector whose each component *h_it_* denotes the number of trees cut from size class *i* at time *t*. This formulation allows the forest owner to have partially conflicting objectives, such as attaining a particular revenue and maintaining a certain biological condition for the stand. In particular, the forest owner benefits both from the revenues from harvesting, so that he tends to harvest more, as well as from the amenities provided by a richer stand, so that he tends to preserve the stand’s growth. He therefore faces a tradeoff between the two objectives. Hence, he/she will favor one or the other based on the resulting optimal harvesting rule, and, consequently, on his/her own degree of uncertainty aversion.

### 2.1 Growth dynamics

Let us consider a simple growth and yield model for an uneven-aged forest stand, relying on the matrix model of uneven aged forest management of Michie and Buongiorno (1984). Uneven-aged forest are characterized by the coexistence of many trees of different age and size on small tracts of land, however, trees are generally grouped in patches of similar age, which are usually too small to be administered as even-aged compartments. In uneven-aged forest, there are always trees left on each hectare, even immediately after harvest. Regeneration usually comes from the stock of saplings in the understory emerging through the openings left by cutting the large trees. Therefore, this form of management works best with trees that are shade tolerant, further, it is very attractive for forests managed for multiple uses, including recreation. The model we present here deals with an uneven-aged stand. The stand we consider should be treated as a unit because it has uniform land quality, topography, and species composition. As in Michie and Buongiorno (1984), the state of a stand is described by the size distribution of trees on an average hectare.

For simplicity, let us assume that there are only three tree size-classes characterizing the stand. As before, we denote by the vector *y_t_* the general stand state at a given point in time *t*, where *y_it_* is the number of trees per acre in size-class *i* at time *t*. Over time, the stand state changes because some trees die, some are cut, some advance to a larger class, and new trees appear in the smallest size class. The stand growth model is a set of equations that predicts the state of the stand at time *t + 1*, given its current state. The time from *t* to *t + 1* is a fixed unit. *a_i_*, is the fraction of live trees in size class *i* at time *t* that are still alive and in the same size class at time *t + 1*. *c_i_* is the fraction of live trees in size class *i* at time *t* that are still alive and have grown into size class *i + 1* at time *t + 1*. The time unit used is short enough that no tree can skip one size class. Consequently, the fraction of trees in age class *i* at time *t* that are dead at time *t + 1* is *1-a_i_-c_i_*, because a tree can only remain in the same class, grow into a larger class, or die. Therefore, the tree-size distribution Table 1 and the equation for ingrowth fully describe the state of the stand at the time in which the inventory is conducted.

**Table 1:** Tree size distribution matrix.

Size class is	Proportion of staying *a_i_*	Proportion of growing up *c_i_*	Proportion of dying *1-a_i_-c_i_*
1	*a_1_*	*c_1_*	*1-a_1_- c_1_*
2	*a_2_*	*c_2_*	*1-a_2_- c_2_*
3	*a_3_*	*0*	*1-a_3_*

Following Michie and Buongiorno (1984), we assume that ingrowth at time *t*, *R_t_ = R(B_t_,N_t_)*, namely the expected number of trees entering in the smallest class during the time-period under consideration, is a linear function of a constant *W* , the stand basal area *B_t_*, and the number of trees after harvesting *N_t_ = y_1t_ + y_2t_ + y_3t_* –(*h_1t_ + h_2t_ + h_3t_*) standing at time *t.*

In particular, *R_t_ = W-r_1_B_t_ + r_2_N_t_*, and *r_1,_ r_2_ > 0*. Where *B_t_ = b_1_(y_1t_-h_1t_) + b_2_(y_2t_-h_2t_) + b_3_(y_3t_-h_3t_)*. Each coefficient *b_i_* is the basal area of the average tree in the corresponding size class.^3^

Denoting as before by *h_it_* the number of trees cut from size class *i* at time *t*, and by *h_t_* the corresponding vector, representing the entire harvest at time *t*, the stand growth model is a set of equations that predicts the evolution of the stand from time *t* to time *t + 1*. There is one equation for each size class:

$y_{1,t+1}=a_{1}\left(y_{1,t}-h_{1,t}\right)+R_{t}=a_{1}\left(y_{1,t}-h_{1,t}\right)+W-B_{t}r_{1}+N_{t}r_{2}$=

=$a_{1}\left(y_{1,t}-h_{1,t}\right)+W-r_{1}\left[b_{1}\left(y_{1,t}-h_{1,t}\right)+b_{2}\left(y_{2,t}-h_{2,t}\right)+b_{3}\left(y_{3,t}-h_{3,t}\right)\right]+r_{2}\left[\left(y_{1,t}-h_{1,t}\right)++\left(y_{2,t}-h_{2,t}\right)+\left(y_{3,t}-h_{3,t}\right)\right]$

$$y_{2,t+1}=c_{1}\left(y_{1,t}-h_{1,t}\right)+a_{2}\left(y_{2,t}-h_{2,t}\right)$$

$$y_{3,t+1}=c_{2}\left(y_{2,t}-h_{2,t}\right)+a_{3}\left(y_{3,t}-h_{3,t}\right)$$

Collecting terms, and using matrix notation, the stand dynamics can be described through a linear state transition law. Setting:

$$D=\left[\begin{array}{ccc} a_{1}-r_{1}b_{1}+r_{2} & -r_{1}b_{2}+r_{2} & -r_{1}b_{3}+r_{2}\\ c_{1} & a_{2} & 0\\ 0 & c_{2} & a_{3} \end{array}\right]$$

and *E = −D*, we can write the stand dynamics of the approximating model as $y_{t+1}=Dy_{t}+Eh_{t}+W1$, where ***1*** *= [1,0,0]^T^*,^4^

The framework presented above does not consider neither risk, nor uncertainty. In reality, many random factors could affect the dynamic growth of the stand. Thus, let us enrich the approximating growth equation describing the stand dynamic as follows:

(1) $$y_{t+1}=Dy_{t}+Eh_{t}+W1+C\varepsilon_{t}$$

where $\varepsilon_{t}$ is an external disturbance vector process. Notice that the term $C\varepsilon_{t}$ allows class-specific shocks for all size-classes and, potentially, depending on the specific characterization of the matrix *C*, also for cross-classes effects, meaning that the shock of a specific *i*-class, $\varepsilon_{i}$, can also affect another size class *j*. The disturbance $\varepsilon_{t}$ is only known to be bounded in some measure, but otherwise unknown. The set of possible disturbances is denoted by ∑. The size of the set ∑ expresses the concern for uncertainty (or robustness) of the forest owner. The larger (smaller) the set, the more (less numerous) are the alternatives to the approximating model considered, the more (less) the uncertainty averse the forest owner is.

To simplify notation, from now on we will omit the termW1 since this can be easily included in a transformation *D’* of the matrix *D*, that describes the dynamics of a vector identical to *y* augmented with one additional state. Therefore, the transformed dynamics is $y_{t+1}=D\text{'}y_{t}+Eh_{t}$, where the transformation D’ is:

***D’=***$\left[\begin{array}{cc} \left[\begin{array}{ccc} a_{1}-r_{1}b_{1}+r_{2} & -r_{1}b_{2}+r_{2} & -r_{1}b_{3}+r_{2}\\ c_{1} & a_{2} & 0\\ 0 & c_{2} & a_{3} \end{array}\right] & \begin{array}{c} W\\ 0\\ 0 \end{array}\\ \left[\begin{array}{ccc} 0 & 0 & 0 \end{array}\right] & 1 \end{array}\right]$ and *E = -D’*.

*y_t_* and *h_t_* have been transformed as *y_t_ = [y_1t_,y_2t_,y_3t_,1]^T^* and *h_t_ = [h_1t_,h_2t_,h_3t_,0]^T^*

### 2.2 Optimization problem without uncertainty

When the forest owner considers neither risk nor uncertainty, he/she will assume that the dynamics of the stand is described by the equation $y_{t+1}=D\text{'}y_{t}+Eh_{t}$, wherein external disturbances are not included. In this case, it is possible to define the expected value of the utility of the forest owner, as he/she knows the probabilities of any occurrence affecting the forest and he acts based on that information. Henceforth, given the growth dynamics for $y_{t+1}$ and the objective function of the forest owner, the corresponding expected utility maximization problem is

(2) $$\max_{h_{t}}E_{0}\sum_{t=0}^{\infty }\beta^{t}u\left(y_{t},h_{t}\right),\;\text{0<}\;\beta \text{< 1}$$

subject to $y_{t+1}=Dy_{t}+Eh_{t}$, given the initial state *y_0_* and with *β* being an intertemporal discount factor such that 0 < *β < 1*. $E_{0}$ denotes expectations at time *t = 0*.

### 2.3 Concern for risk and robustness

External factors might significantly affect forest growth and the provision of ecosystem services. However, neither the degree of the resulting shock nor the reaction of forest ecosystems are known in advance, leading to considerable uncertainty.

To consider such uncertainty, we assume that a representative forest owner regards his model $y_{t+1}=D\text{'}y_{t}+Eh_{t}$ only as an approximation to an unknown true forest growth model, wherein external disturbances are included. A true plausible model is described as:

(3) $$y_{t+1}=D^{\text{'}}y_{t}+Eh_{t}+\varepsilon_{t}$$

where $\varepsilon_{t}$ belongs to a set ∑ with elements all the possible disturbances that the forest owner considers plausible, including the case of no-disturbance, that is, 0∈∑ (notice that with the exclusive purpose of simplifying the notation, we have set to the identity matrix the matrix *C*). In particular, ∑ is a set of vectors $\varepsilon_{t}$with components $\varepsilon_{\mathrm{it}}$, where $\varepsilon_{\mathrm{it}}$ represents a shock at time *t* to age class *i*, which the forest owner/manager considers plausible at the moment of the harvest decision. The forest owner constructs his own set of conjectures ∑ by collecting and evaluating information. Shocks outside the set ∑ are considered implausible, and therefore ignored. This of course does not exclude that the forest owner might have been wrong *a posteriori* and the realized shock is actually outside ∑.

Hence, the forest owner believes that forest dynamics are described by equation (3), for some unknown process $\varepsilon_{t}$in the set ∑. The size of the set ∑ is essentially a measure of the discrepancy between the approximating and the true model, as a smaller ∑ implies that the two models, with and without external disturbances, are difficult to distinguish from one another. In what follows, we will only consider forest owners who are either neutral (i.e., ∑ is a singleton whose only element is 0) or averse to model uncertainty. We will equivalently say that forest owners are (not) concerned with model uncertainty if they are averse (neutral) to model uncertainty.

A forest owner averse to uncertainty wants to establish harvesting rules robust to model misspecification, meaning that they work well over a set of models of the form (1) under the constraint $\varepsilon_{t}$∈∑.

The standard way to accommodate for this quest for robustness is to consider worst-case scenarios, which translates into solving a minimax problem, that is:

(4) $${\min_{\varepsilon }max}_{h_{t}}E_{0}\sum_{t=0}^{\infty }\beta^{t}u\left(y_{t},h_{t}\right),$$

subject to $y_{t+1}=D\text{'}y_{t}+Eh_{t}+\varepsilon_{t}$ and $\varepsilon_{t}$∈∑.

The forest owner/manager is concerned about misspecification of the growth model and therefore seeks a harvesting rule that will work well across a set ∑ of models surrounding his approximating one. In particular, he wants to derive a single harvesting rule that will work reliably well for all models in the set ∑. At mathematical level, robust control theory solves this problem by finding a robust harvesting decision by using a Bellman equation in which the forest owner/manager maximizes his intertemporal objective over feasible harvesting, rules while a hypothetical malevolent nature minimizes that same objective by choosing the shock to the growth model. The malevolent nature is just a device used to construct a lower bound on the performance of the harvesting rule. A closed solution to the problems above (with and without uncertainty) cannot be easily found, but it can still be derived using an appropriate optimization software.

It should be noted that we implicitly assume that shocks are group-size and time independent. Removing this assumption could exceedingly complicate the optimization problem, leading to unfeasibility. In the section that follows, we will therefore conduct a numerical experiment to compare optimal harvesting rules with and without a concern for uncertainty.

## 3 Numerical analysis

We present a numerical exercise to show a concrete application of our framework and to evaluate the effects of uncertainty. For simplicity, we use the same numerical example as in Buongiorno and Gilles (2003), whose parameters are based on observations from permanent plots in sugar maple stands in Wisconsin. As mentioned above, we consider three size (diameter) classes. The framework can, however, be easily extended to any possible dimension. Further, it is flexible enough to be enriched by other elements, such as market prices, harvesting costs, and alternative utility specification. There are three types of representative forest owners. One who is not concerned with model uncertainty, while two others are concerned to different degrees (low and high, respectively, or, equivalently, Low UA and High UA). We consider their behavior, assuming identical forest stand endowments, summarized in Table 2, Table 3.

**Table 2:** Tree distribution.

*Diameter class*	*Diameter range (cm)*	*Number of trees (/ha)*	*Average diameter (cm)*	*Basal area of average tree (m^2^)*	*Total basal area (m^2^/ha)*
*1*	*10*–*19.9*	*840*	*15*	*0.02*	*14.8*
*2*	*20*–*34.9*	*234*	*27*	*0.06*	*13.4*
*3*	*35+*	*14*	*40*	*0.13*	*1.8*
*Total*		*1088*			*30.0*

**Table 3:** Proportion of trees staying in the same class/growing/dying within 5 years.

*Size class is*	*Proportion of staying a_i_*	*Proportion of growing up c_i_*	*Proportion of dying 1-a_i_-c_i_*
*1*	*0.80*	*0.04*	*0.16*
*2*	*0.90*	*0.02*	*0.08*
*3*	*0.90*	*0.00*	*0.10*

In uneven-aged stands, the number of trees per unit area decreases progressively as the size of the trees increases. The basal area of an average tree in each size class, is the area of the cross section of the tree, measured at breast height. In Table 3, a_1_ = 0.80 and c_1_ = 0.04 mean that 80% of the trees in the smallest size class were in the same class at time t + 1, while 4% of the trees in the smallest size class grew into the larger class. The remaining 16% died.

Following Buongiorno and Michie (1980), we set in *R_t_ = W-r_1_B_t_ + r_2_N_t_ r_1_* to *−9.7* and *r_2_* to *0.30*, and the coefficients *b_1_*, *b_2_* and *b_3_* to *0.02*, *0.06* and *0.13*, respectively in *B_t_ = b_1_(y_1t_-h_1t_) + b_2_(y_2t_-h_2t_) + b_3_(y_3t_-h_3t_)* (Table 2). Therefore, using the coefficients in Table 2 and the expression for the matrix D above, we can derive:^5^

$$D=\left[\begin{array}{ccc} 0.92 & -0.29 & -0.96\\ 0.04 & 0.90 & 0\\ 0 & 0.02 & 0.90 \end{array}\right]$$

We consider 50 years (i.e., 10 time steps). At each time step, a particular shock could potentially affect the forest, meaning that the growth in each class could be different from the expected one.

We let the initial condition of the stand to be described by *y_0_ = [840,234,14]’*. The forest owner is a price taker and, given wood prices available on the market, he has computed the initial harvest level as *h_0_ = [168,35.1,1.4]’* . Thus, *h_0_* corresponds to the cutting rule taking 20% of the smallest trees, 15% of the mid-size trees, and 10% of the largest trees. We will also set the constant *W* for the recruitment to 168. The harvesting rule and the hypothesis that recruitment is aimed at reintegrating harvest from the youngest class (hence *W* = 168) come from Buongiorno and Michie (1980). Indeed, here we are mainly interested in qualitatively comparing the harvesting behavior of forest owners with different degrees of uncertainty aversion, the quantitative results per se are less interesting. The forest owner has two conflicting objectives. On one side, he/she wants to maximize the volume of his/her stand to enrich the benefits deriving from the available amenities and ecosystem services. On the other side, he/she wants to maximize the harvest level from each size-group to earn profits. The time horizon considered is 50 years, and no final condition is set.

This particular formulation in terms of objective function is interesting, as it allows for a trade-off between the realization of a particular harvest level and the attainment of a specific distribution for the forest stand. It is of interest to analyze whether forest owners will favor one objective over the other depending on their level of aversion to model uncertainty. We would like to emphasize that our model per se does not restrict the choice of the objective function. Similarly, in this exercise, we have adopted a number of simplifications/restrictions, which are not strictly requested by our setting. Specifically, we have set to the unit the price of harvest from each size group, and we have not considered harvesting costs. Implicitly, we have also assumed that the economic value for amenities/ecosystem services from a certain forest area is in 1:1 relation with the growing stock of the area in question. We want to emphasize that here we are only showing how our framework could be applied. Further, as our focus is uncertainty, we did not want to introduce any other element that could possibly impair the interpretations of our results.

The optimization problem of the forest owner consists in choosing the optimal harvest level *h* to minimize

(5) $$E_{0}\sum_{t=1}^{N}\beta^{t}\left(y_{t}+h_{t}\right)$$

under the dynamics described above, and the initial levels *y_0_* and *h_0_*. We set N to 10 and β to 0.97, the exact values of these two parameters do not affect the qualitative relations and results of our analysis.

Next, we consider three alternative harvesting behaviors, one corresponding to a forest owner who does not consider model uncertainty, and two corresponding to two forest owners who consider model uncertainty, but to different degrees. The two optimization problems, with and without the concern for model uncertainty are respectively:

(6) $$\max_{h}E_{0}\sum_{t=1}^{N}\beta^{t}\left(y_{t}+h_{t}\right)$$

subject to $y_{t+1}=Dy_{t}+Eh_{t},$ given the initial state *y_0_* and harvest level *h_0_*

and

(7) $${\min_{\varepsilon }max}_{h}E_{0}\sum_{t=1}^{N}\beta^{t}\left(y_{t}+h_{t}\right)$$

subject to $y_{t+1}=Dy_{t}+Eh_{t}$**,** given the initial state *y_0_* and harvest level *h_0_*

In the following, the label *Neutral* will identify the agent who is not concerned with model uncertainty and fully trusts the model. The other labels, *Low UA* and *High UA,* identify forest owners that are concerned with model uncertainty, with a low and high, respectively, level of aversion. Next, we need to define the ambiguity sets for the ambiguity averse agents. In the analysis that follows, we are not really interested in quantitatively evaluate the effect of uncertainty, but rather to qualitatively describe the effects of increases in such uncertainty. It is therefore essential to define the ambiguity sets of *Low UA* and *High UA*, so that the two forest owners are comparable in terms of ambiguity aversion, and, in particular, *Low UA* is less averse than *High UA*. This implies that the ambiguity set of *Low UA* should be a subset of the one of *High UA*. The simplest way to ensure this is to consider two plausible (positive or negative) percentage variations from the initial stock, with the one of *Low UA* being smaller in absolute value than the one of *High UA*. Thus, for simplicity, we will assume that the *Low UA* considers as plausible variations from the reference model of up to 10% from the initial stock, while the *High UA* of up 15%. We are not assuming that only negative shocks are plausible, in this way we can consider all types of uncertainty, possibly including the positive effects on growth resulting from improved planting materials, or those related to climate change, as well as the possible (negative) damages on forests. Hence *Low UA* (*High UA*) agent believes that the time *t* shock ε_1t_ in the largest group will be such that −8.40(−12.6) < ε_1t_ < 8.40(12.6). Similarly, the time *t* shock ε_2t_ in the second group is believed to be such that-2.34(-3.51) < ε_2t_ < 2.34(3.51), and ε_3t_ in the third group −0.14(-0.21) < ε_3t_ < 0.14(0.21). Again, for simplicity, we assume that these bounds are fixed for any *t*, even if this is not a strict requirement of the model. We want to emphasize that any other selection of these thresholds would have worked, indeed here we are only interested in qualitative comparison among the different attitudes of agents with a different degree of uncertainty aversion, and not in quantitatively asses their harvesting behavior. Hence we set:

(8) $$\sum lowUA=\left\{{-8.40\leq \varepsilon }_{1}\leq 8.40,{-2.34\leq \varepsilon }_{2}\leq 2.34,-{0.14\leq \varepsilon }_{3}\leq 0.14\right\}$$

and$\sum highUA=\left\{{-12.60\leq \varepsilon }_{1}\leq 12.60,{-3.51\leq \varepsilon }_{2}\leq 3.51,-{0.21\leq \varepsilon }_{3}\leq 0.21\right\}$. (9)

For the numerical analysis, we use MATLAB integrated with Yalmip (Lofberg, 2004). Yalmip is a numerical toolbox for modeling and optimization in MATLAB, which is currently freely available online (https://yalmip.github.io).

## 4 Results and discussion

There are notable differences in harvesting behavior between the different owner types. The harvesting schedules of the three forest owner types for the entire time-horizon (50 years) are reported in Table 4.

**Table 4:** Harvest levels by forest owner type over tree diameter-class and five-year period.

	Tree diameter-class	h_1_	h_2_	h_3_	h_4_	h_5_	h_6_	h_7_	h_8_	h_9_	Total over time
Neutral	3	0.00	0.00	0.00	0.00	0.00	0.00	0.00	0.00	275.17	275.17
	2	228.45	24.30	1.64	0.00	17.53	33.27	0.00	10.84	23.71	339.73
	1	14.00	0.00	0.17	0.21	0.00	0.16	0.46	0.20	0.00	15.21
LowUA	3	0.00	0.00	0.00	0.00	0.00	0.00	0.00	0.00	155.09	155.09
	2	224.37	19.83	0.52	0.00	15.16	21.05	0.00	6.00	19.83	306.77
	1	13.70	0.00	0.00	0.17	0.00	0.01	0.12	0.00	0.00	14.01
HighUA	3	0.00	0.00	0.00	0.00	0.00	0.00	0.00	0.00	90.72	90.72
	2	222.30	17.62	0.00	0.00	13.94	9.54	0.00	2.97	22.17	288.54
	1	13.45	0.00	0.00	0.08	0.00	0.00	0.00	0.00	0.10	13.63

Model uncertainty should affect larger classes more severely than the smallest tree diameter-class, as in this case uncertainty is reduced by the constant non-stochastic ingrowth (smaller trees are here synonymous with younger trees). For the same reason, also the difference between the two uncertainty averse agents, as well as those between each of the averse agents and the neutral one, should be more pronounced for larger classes. These tendencies are immediately confirmed when comparing the levels of total harvest for each tree diameter-class: uncertainty averse agents, harvest less than uncertainty neutral ones, in particular for larger classes. Indeed, for larger classes, there is less of a possibility to revert the negative tendency of a possible (negative) shock in the future while the small tree diameter- class experiences ingrowth in any period, reducing the perceived ambiguity. A LowUA (HighUA) agent harvests 44% (67%) less than the uncertainty neutral one from the third class, 10% (15%) from the second class, and only 8% (10%) from the smallest class. In addition, the difference between the uncertainty averse agents is more perceivable for the larger tree class, as the LowUA harvests 42% more than the HighUA, while for the other two classes he/she harvests 6% and 3% more from second and first class, respectively.

**Fig. 1:**
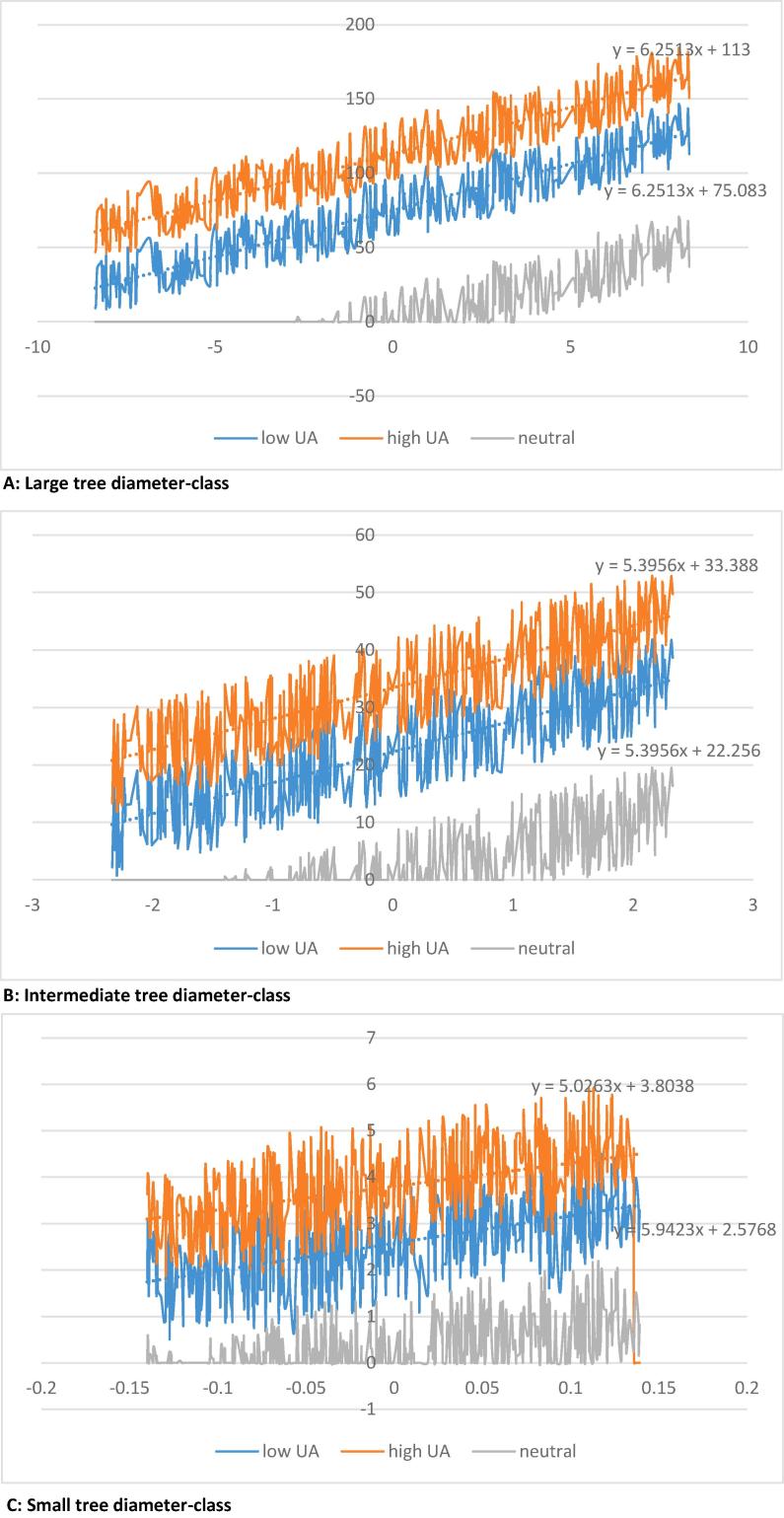
Simulation of the growing stock (vertical axes) after 50 years for the three owner types (COLOR) for specific possible realizations of the class specific shock (horizontal axes).

We simulate the possible evolution of forest stands, after the realization of the shock, for demonstration purposes only. We assume that the shock realized at time 0 (ε_0_) will persist over time, i.e., ε_0_ = ε_t_ = ε_t+1_, even if none of the three forest owners is aware of it. We simulate 500 values for the shock in each tree size class, within the bounds considered by the low UA agent (i.e., belonging to the set ∑_lowUA_). As the decision is taken at time zero, the harvest levels are those reported in Table 4 for each agent. The three plots of Fig. 1 reports, for each particular size-diameter class (that is, larger, intermediate and smaller, to each of which it corresponds one plot), and for each owner type (blue for *Low UA*, grey for *neutral* and orange for *High UA*), on the vertical axes the simulated growing stock after 50 years of that particular size class, as a function of the class specific shock, whose intensity is measured on the horizontal axes.

The growing stock is markedly higher for ambiguity averse forest managers for all tree size classes at the end of the time horizon, i.e., after 50 years (Fig. 1), reflecting lower harvest levels of forest managers concerned with model uncertainty, favoring stand volume over harvest revenues. In particular, notice that for low occurrences of the shocks the stand in the two larger tree size-classes is fully depleted for the uncertainty neutral agent. In general, also for positive occurrences, the final growing stock is considerably smaller for the neutral agent.

As the three class-specific shocks are independent, it is not possible to compare the utility of the three agents. Nevertheless, something more can be said about the two uncertainty averse agents, at least for what concerns the average utility deriving from the two larger tree classes. Indeed, the lines describing the tendencies of the data from the simulations related to the two uncertainty averse agents are parallel for the two larger diameter classes. In particular, the growing stock in these two classes is larger for the high UA agent, approximately for any possible realization of the shock in the set ∑. Thus, the loss at the end of the 50 years in terms of utility of the low UA agent could approximately be measured by the difference between the intercepts of the two tendencies line, specifically −37.917 (that is, 75.083–113) and −11.132 (that is, 22.256–33.388), for the larger and the intermediate class, respectively. This loss is more than compensated by the higher harvest levels at the end of the 50 years from these two groups (64.37 more from the larger group and 18.23 from the intermediate one), as the low UA agent has harvested more due to his higher degree of confidence in the model.

### 4.1 The role of information

The restriction $\varepsilon_{t}$ ∈∑ on the specification of the model governing the growth process has intuitive interpretation in terms of information release. Thus, one could easily imagine a policy maker that shares his/her (approximating) forest growth model with forest owners /managers, simultaneously making them aware that this is only a good approximation of the true model. The true model includes an additional shock $\varepsilon_{t}$, whose size is restricted by theset∑. The more precise the information, the smaller is the set of alternative specifications to be considered, ∑.

The results above could thus also be interpreted in terms of forest managers differing as to the access to information, specifying the set of alternative models to be considered by means of two different ambiguity sets, ∑_LUA_ and ∑_HUA_, with $\sum_{\mathrm{LUA}}includedin\sum_{\mathrm{HUA}}$. Hence, the set of alternative specifications considered by the agent *LUA* is a subset of the one considered by the *HUA* agent, in other words, *LUA* is more informed. Consequently, the simulation results clearly suggest that issuing information can affect harvesting behavior and forest resource development in the context of model uncertainty, as forest owners vary their chosen harvest levels depending on whether they have access to a less (∑_HUA_) or more (∑_LUA_) precise set of information. This is consistent with findings by Sousa-Silva et al (2016) regarding the importance of information for climate change related actions of forest managers. In the context of our example, a policy maker willing to increase (reduce) harvest levels from all tree diameter-classes should release more (less) precise information, inducing agents to consider a smaller (larger) set of alternative model specifications $\sum_{\mathrm{LUA}}$ ($\sum_{\mathrm{HUA}}$).

## 5 Summary and conclusions

We introduce a theoretical framework accounting for model uncertainty. Forest owners/managers in the framework regard their decision support system only as an approximation to an unknown, true, model. This specific theoretical representation is particularly relevant in view of forest management uncertainty induced by climate change. Indeed, the understanding of the magnitudes of climate change—if not the general direction— is still rather weak, and even more so when it comes to the reaction of forest ecosystems to climate change (see, e.g., Belyazid and Giuliana, 2019, Subramanian et al., 2019). In our analysis we are not assuming as plausible only negative shocks to forests and the demand for forest products and services, instead all types of uncertainty are considered, e.g., related to unforeseen positive forest growth effects from climate change (CO2 fertilization, longer vegetation periods) as well as positive demand shocks.

Our results show that the degree of perceived uncertainty of forest owners/managers as to the accuracy of their decision support system, along with their level of uncertainty-adverseness, affects harvesting behavior and forest development. Therefore, also the perceived level of uncertainty resulting from the release of information affects harvest behavior and forest development. The forest owner we consider has partially conflicting objectives, as he/she tries to maximize revenues but also to increase stand volume. The simulation shows that the tendency to favor one objective with over the other depends on the degree of uncertainty and uncertainty aversion, as well as the level and quality of information received. Thus, information release alleviating the uncertainty perceived by forest owners would result in a change of priorities between harvesting revenues and the preservation of forest stand characteristics (the latter amenities here approximated by the growing stock). This highlights the importance of information as a policy instrument in the context of model uncertainty.

It should be underlined that the suggested framework is intrinsically static, in that the harvesting schedule is established at the beginning of the period based on the current realization of the shock, with no updating regarding neither the future harvesting schedule nor the available information and the ambiguity set to be considered. Relaxing this assumption is an interesting and important point, which we leave for future research.

## CRediT authorship contribution statement

**Francesca Rinaldi:** Conceptualization, Methodology, Software, Formal analysis. **Ragnar Jonsson:** Writing - original draft, Writing - review & editing.

## Footnotes

1 Gilboa and Schmeidler (1989) discuss how a max–min rule smooths, or averages, utility distributions over the set of plausible odds, thus making the decision maker better off. This special characterization can be understood imagining a malevolent nature that wants to minimize the same objective as the decision maker. Hence, the problem reduces to a zero-sum game. In this type of games, a person can win only if the other player loses, so that the sum of the two payoffs is exactly zero. Thus one player’s strategy is to *maximize* his own *minimum* gain, while the other player’s strategy is to *minimize* his own *maximum* loss. Thus, given malevolent nature’s move, the decision maker tries to maximize the objective function using certain control-decision variables. At the same time, given the decision maker’s move, the malevolent nature tries to minimize it, choosing the probability distribution over the probabilities space, hence the maxmin characterization.

2 This is clearly a simplification. However, it is necessary only for comparative purposes of the results among different agents.

3 The simplest option would be to assume a constant R_t_. However, even though recruitment is quite erratic, it is influenced by the stand state. Michie and Buongiorno (1980) found that, at least for sugar maple, recruitment was affected mostly by the stand basal area and by the number of trees per hectare. Michie and Buongiorno (1980) estimates econometrically the parameters of the equation for *R_t_*, we use their parameters also here. To not loose generality, in our model, the constant *W* is linked to the initial harvest level and not to the specific value found in Michie and Buongiorno (1980) for sugar maple. Indeed, general evidence (Michie and Buongiorno (1980)) suggests that recruitment is aimed at reintegrating harvest from the youngest class.

4 The resulting product W1 is just a column vector with first component equal to *W* and the other two to 0.

5 All parameters in Buongiorno and Michie (1980) are based on observations from permanent plots in sugar maple stands in Wisconsin.
